# Evaluation of a Centrifuged Double Y-Shape Microfluidic Platform for Simple Continuous Cell Environment Exchange

**DOI:** 10.3390/ijms13010819

**Published:** 2012-01-13

**Authors:** Akihiro Hattori, Kenji Yasuda

**Affiliations:** 1 Kanagawa Academy of Science and Technology, KSP East 310, 3-2-1 Sakado, Kawasaki 213-0012, Japan; E-Mail: hattori.bmi@gmail.com; 2 Department of Biomedical Information, Institute of Biomaterials and Bioengineering, Tokyo Medical and Dental University, 2-3-10 Kanda-Surugadai, Chiyoda, Tokyo 101-0062, Japan

**Keywords:** microfluidic chip, Y-shape platform, medium exchange, environmental control, single cell, centrifugal force, on-chip cellomics

## Abstract

We have demonstrated the efficacy of a microfluidic medium exchange method for single cells using passive centrifugal force of a rotating microfluidic-chip based platform. At the boundary of two laminar flows at the gathering area of two microfluidic pathways in a Y-shape, the cells were successfully transported from one laminar flow to the other, without mixing the two microfluidic mediums of the two laminar flows during cell transportation, within 5 s with 1 *g* (150 rpm) to 36.3 *g* (900 rpm) acceleration, with 93.5% efficiency. The results indicate that this is one of the most simple and precise tools for exchanging medium in the shortest amount of time.

## 1. Introduction

In many areas of cell-based screenings such as blood monitoring, process control in pharmaceutical fermentations, continuous real-time monitoring of biological agents is of the utmost importance. The key requirement to perform such monitoring tasks is the fast and precise exchange processing of cell environments such as buffer exchange. Recently, microfluidic platforms [1,2] for the continuous separation of biomolecules and particles have been proposed. Some of these use passive working principles [3–5], others apply dielectrophoresis [6,7], acoustic standing waves [8–11], segmented flow [12–14], or magnetophoresis [15,16]. Applying centrifugal force is one of the most powerful candidates for the passive steady-non pulsatile-flow in microfluidic systems [17] for continuous fully automated processing such as cell arrangement [18], DNA concentration [19], biomolecular separation [20], and also a compact disc (CD) microfluidic chip based enzyme-linked immunosorbent assay (ELISA) [21], whole blood glucose analysis [22], and hybridization assay for phenylketonuria (PKU) screening [23]. Especially, extraction of plasma from whole blood exploiting microfluidic pathways with centrifugal pumping [24] and cell trapping exploiting reagent-impregnated agarose-made micro holes with centrifugal liquid pumping [25] were examined for practical applications. Yet the majority of the proposed platforms are at the proof-of-concept stage of the system integration, and none of them have examined the ability of microfluidic platforms for quick exchange of cell environments in single cell level.

Therefore, in this paper, we have evaluated the efficacy of the exchange of environments of single cells within the simple Y-shape microfluidic chip with passive centrifugal force with charge coupled device (CCD) camera image analysis.

## 2. Results and Discussion

### 2.1. System Design

**Figure 1:**
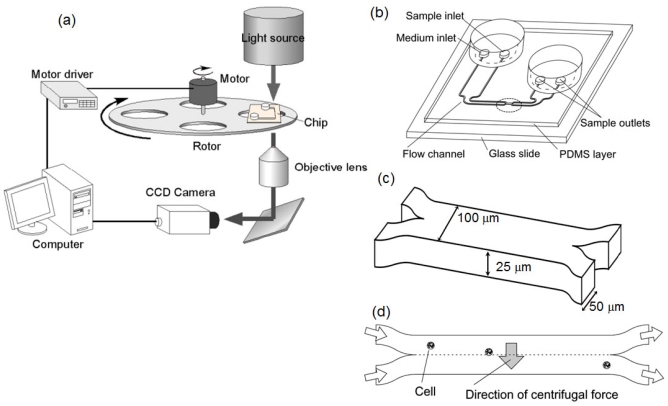
Schematic view of the system setup and microfluidic structure of microfluidic chip. (**a**) System setup; (**b**) microfluidic chip design; (**c**) and (**d**) gathering part of double Y-shape microfluidic pathway.

The continuous exchange of a medium condition surrounding single cells has been performed by the following three processing steps: cell introduction into the Y-shape microfluidic pathway, a medium exchange step that removes the environmental medium buffer by cells’ shift from one laminar flow to the other through the boundary of two laminar flows, and cell collection by another reverse-Y-shape microfluidic pathway. On our microfluidic platform the above three steps are implemented in a microfluidic structure which is contained in a monolithic chip. A rotating platform provides the required passive centrifugal force field for cell motion within the chip. For the constant centrifugal force generation, a rotating stage with a stepping motor (TAMAGAWA SEIKI, 5PHASE STEPPING MOTOR 02K-S523W-∑) and a control unit (SIGMA KOKI, Stage controller SC-101G) was mounted in the optical microscope system (OLYMPUS IX70) [Figure 1(a)].

The microfluidic chip comprises five parts [Figure 1 (b)]: (1) two inlets (inner one is for sample cell solution and the other outer one is for the washing buffer); (2) Y-shape microfluidic pathway (flow channels) guiding two buffers into the channel junction; (3) the channel junction where two laminar flows are brought into contact via a laminar-flow interface (boundary) in order to transfer the cells from one buffer to the other; (4) reverse Y-shape pathway to split into two laminar flows once again; and (5) two outlets (inner one is for sample cell buffer and the other outer one is for the cells with washing buffer). The 2D-layout of the microfluidic structure was fabricated in the polydimethylsiloxane (PDMS) [26,27] attached to a 1-mm-thick glass slide.

### 2.2. Rotation of Chip for Centrifugal Force Generation

The rotating stage was set at the position of the focal plane of the microscope, and the chip was set on the stage (4 cm from the center of rotation), providing sufficient centrifugal force for cells transportation inside the microfluidic chip. The role of centrifugal force in this system was for two purposes: to generate the driving force for injecting buffers into the microfluidic flow with the same pressure and velocity; and secondly to transport cells from one laminar flow to the other.

**Figure 2:**
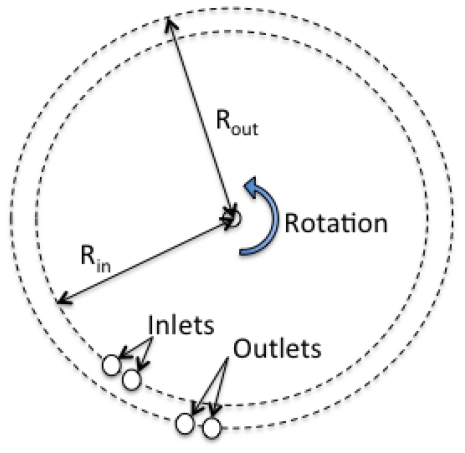
Schematic images explaining spatial arrangements of chip inlets/outlets arrangements and chip rotation for centrifugal force generation.

In order to maintain the two laminar flows at the junction area, the velocity must be synchronized precisely at this area. In this system, each of the two inlets and two outlets were designed at the same distance from the center of rotation (Figure 2). The acceleration in microfluidic chip was determined by *rω^2^*, where r is the distance of the chip from the center of rotation (4 cm), *ω* is angular velocity of rotator. In this case, the accelerations were 1 *g* for 2.5 Hz rotation, and 36.3 *g* for 15 Hz.

### 2.3. Cell Motion in Microfluidic Chip Under Centrifugation

**Figure 3:**
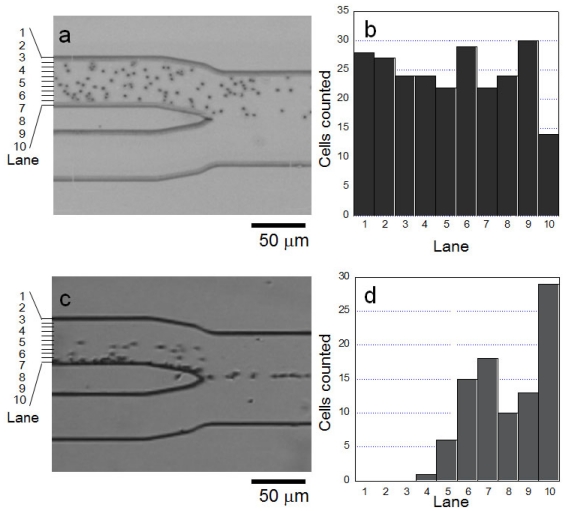
Effect of centrifugal force on erythrocytes in the microfluidic chip, (**a**) micrograph of erythrocyte distribution before centrifugation started; (**b**) spatial distribution of erythrocytes in (**a**); (**c**) micrograph after centrifugation started; and (**d**) spatial distribution in (**c**).

Under the influence of the centrifugal force provided by the rotation of the microfluidic chip, the cells gathered on the outer wall of the microfluidic pathway. As shown in [Figure 3 (a,b)], before centrifugal force was applied, whole erythrocyte cells were dispersed in the pathway. Whereas, once the centrifugal force was applied [Figure 3 (c,d)], whole cells shifted to the outer wall of microfluidic pathway under 1 *g* acceleration condition with a 100 μm·s^−1^ velocity of buffer.

### 2.4. Cell Transportation Between Two Laminar Flows

We checked two topics regarding this centrifugation platform: firstly, the evaluation of cell transportation from one pathway to the other, and, secondly, the evaluation of mixture of two laminar flows during the cell transportation process.

**Figure 4:**
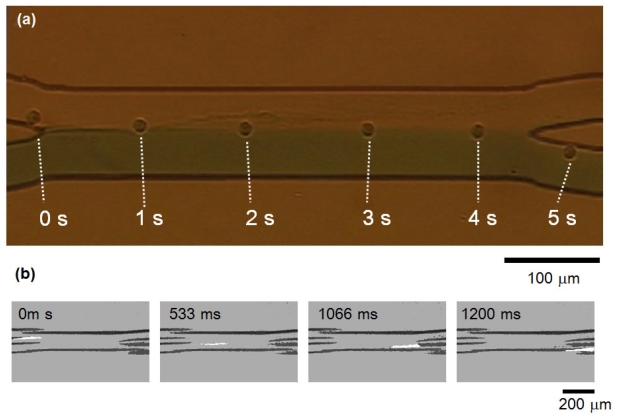
Time course tracking of cell transportation. (**a**) movement of single HeLa cell from one laminar flow to the other under 1 *g* acceleration with 2.5 Hz rotation; (**b**) movement under 36.3 *g* with 15 Hz rotation.

Figure 4 (a) shows the results of the above two subjects, *i.e.*, cell transportation and medium mixture. As shown in this figure, a HeLa cell with a 100 μm·s^−1^ flow velocity, shifted successfully from the first laminar flow to the other along the 400 μm length of two laminar flow boundaries at the gathering area. In contrast, the medium buffers maintained their boundaries, and no mixture was observed either visibly or in fluorescence detection of collected buffer (we added fluorescent dye (Rhodamine B) into one of two buffers for quantitative evaluation of mixture).

We have also checked the cell transportation under stronger centrifugal force conditions. As shown in Figure 4 (b), we added 36.3 *g* (15 Hz) acceleration into the microfluidic chip and confirmed the cells having 200 μm·s^−1^ velocity successfully shifted from one laminar flow to the other, and no fluorescent medium mixture was observed using fluorescence detector.

As this exchange was performed successfully even under 1 *g* centrifugal force condition, that means we can exchange medium buffer continuously using gravity without using centrifugal force. As expected, when we set this microfluidic chip perpendicular to the gravity, the cells were separated effectively with no centrifugal force applied.

**Figure 5:**
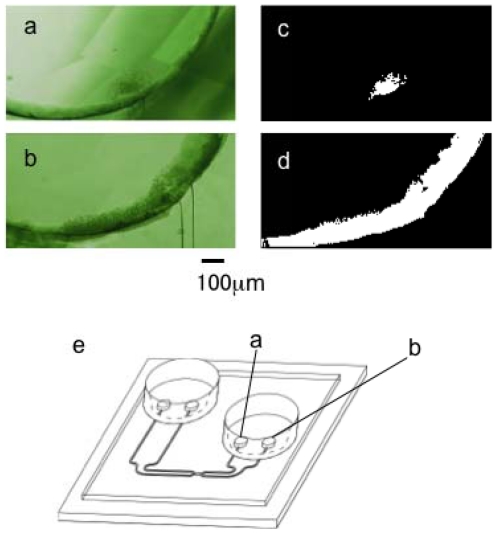
Images of sample outlets after the cell transportation, (**a**) Inner outlet: (**b**) Outer outlet; (**c**) and (**d**) are binary images of cells in (**a**) and (**b**). respectively; (**e**) The positions of outlets.

Figure 5 shows the images of inner outlet (a) and outer outlet (b) after the cell transportation experiment was completed. As shown in the micrographs, most of the HeLa cells were transported to the outer outlet (b), whereas a small amount of cells remained in the inner outlet (a), which might be transported before centrifugal force was generated. Figure 5(c,d) are the binary images of Figure 5 (a,b), and indicate the rough population of cells in those outlets, *i.e.,* the cross sectional areas of (c) and (d) are 211 pixels and 3022 pixels, respectively. Hence, the estimated transportation efficiency in this experiment was 93.5% [=3022/(3022 - 211)].

## 3. Experimental Section

### 3.1. Optical Setup

For the optical observation, a charge-coupled device (CCD) camera (TOSHIBA JK-TU53H) equipped with ×4 objective lens (OLYMPUS. Uplan F1 4×) was used to acquire images and video clips of the cells inside the chip.

### 3.2. Samples

HeLa cell line was obtained commercially (Dainippon Sumitomo Pharma) and were suspended in PBS at a concentration of about 10^4^ -10^5^ cells per milliliter. Erythrocytes were obtained from horse blood and were suspended in physiological saline at a hematocrit of 3.3% for experiments. The cells were suspended in the standard phosphate buffer and applied to the chip. For the washing buffer, the same standard phosphate buffer containing Rhodamine B was applied.

### 3.3. Experiment Procedure

Just before rotation starts, 50 μL of sample cell buffer was applied into the inner inlet, and 50 μL of sample cell buffer was applied into the outer inlet. Then the rotation was started and CCD observation was recorded. After whole buffer at the inlets were transported to the outlets, rotation was stopped and the chip was removed from the rotating stage, and observed the outlets by optical microscope.

### 3.4. Flow Velocity Estimation

Flow velocity in the microfluidic flowchannels were estimated by the measurement of movement of cells in the flowchannels by CCD camera images (frame rate, 1/30 s intervals).

### 3.5. Superimposed Image Acquision and Image Analysis

For superimposed, quantitative evaluation and comparison of images, we used Image J software. The acquired analog CCD camera images were recorded in Digital Tape Recorder (SONY WVD9000), and the recorded data was digitized and stored using image converter of PC (SONY RX-71). The digitized bit map images were then imported Image J software for the further processing.

## 4. Conclusions

In this paper, we introduced the experimental results of a fast and simple cell medium exchange using a double Y-shape microfluidic chip and centrifugal force. Based on the simple rotating platform, we demonstrated that cells can shift effectively from one pathway to the other without any mixture of two laminar flows of different buffers even using 1 *g* acceleration. Using appropriate buffer solutions, the reported platform can be used for continuous automation of quick and single-step minimum buffer exchange in many other well-known processes of cell studies, such as quick exchange of enzymatical treatment buffer without dilution steps or inhibitor additions, quick staining of cells with minimum volume of expensive fluorescent dye, or precise time course measurement of effect of compounds.
